# Phase separation in nuclear biology

**DOI:** 10.1080/19491034.2024.2310424

**Published:** 2024-02-12

**Authors:** Hao Jiang

**Affiliations:** Department of Biochemistry and Molecular Genetics, University of Virginia School of Medicine, Charlottesville, VA, USA

The central mission of the nucleus is the proper maintenance and expression of genetic information. Genes are faithfully duplicated through DNA replication, and damaged DNA needs to be repaired to preserve integrity of the genetic information. Transcription and RNA-processing are two integral parts of expression of genetic information in the nucleus. The proper and dynamic organization of genetic information through control of local and high-order chromatin architecture is the foundation of all these processes. We have achieved remarkable amount of knowledge of the molecular components and the biochemical mechanisms of each of these nuclear processes, but are falling behind in understanding how these processes are spatiotemporally regulated in the very crowded, extremely heterogeneous, and highly dynamic nuclear environment.

Liquid–liquid phase separation has emerged to be a fundamental mechanism in formation of biomolecular condensates that function to organize cellular space and biochemistry. Phase separation is very prominent and plays particularly interesting roles in the nuclear processes. This is in part because the natural long polymers of DNA and RNA can potentially provide multivalent interactions for nuclear proteins, which are also especially enriched for long and intrinsically disordered regions (IDRs) relative to proteins in other cellular compartments. Chromatin can act as central organizational and regulatory platform for nuclear condensate formation and properties, including facilitating their formation but also regulating (often constraining) the mobility and coalescence of nuclear condensates. On the other hand, through selectively gathering macromolecules and their molecular activities in specific compartments with proper composition and dynamics, these condensates profoundly influence the local organization as well as high-order architecture of chromatin and likely all activities associated with chromatin.

This Special Focus collects a total of six articles, including five review articles and one research article, on how phase separation regulates the major nuclear processes in organization, maintenance, and expression of genetic information.

The first set of two reviews focus on the question how phase separation regulates the organization of genetic information in chromatin structure. Cai and Wang [1] review how IDRs widely found in transcription factors and chromatin-modulatory proteins mediate phase separation to regulate high-order chromatin structures and looping. They discuss recent advances in showing how IDR-mediated phase separation can be an important mechanism in shaping the 3D chromatin organization at different scales of genomic length. Phase separation plays a key role in the formation of chromatin loops, such as long-range enhancer – promoter interactions, and can also influence dynamic reorganization of the higher-order structure such as topologically associating domains. They also review a few prominent examples in which the alterations of phase separation lead to disease, especially cancer, through changes in 3D genome organization and dynamic reorganization. Compared to this review [1] that emphasizes the organization of euchromatin more associated with gene activation, the review by Zhang et al. [2] focuses on the role of phase separation in the formation and dynamic regulation of heterochromatin compartmentalization. In part through in-depth discussion of three examples (Xist, MeCP2, and HP1), it summarizes how heterochromatin compartmentalization is regulated by phase separation that is dependent on DNA, histone, and RNA, respectively. This nicely illustrates the central role of chromatin as an organizational and regulatory platform for nuclear phase separation, as mentioned above.

The second set of two papers involve how phase separation plays a role in the proper maintenance of genetic information through regulating DNA damage response and repair. The research article by Wang et al. [3] reports an interesting observation that ciRS-7, a circular noncoding RNA, enhances phase separation of miRNA-induced silencing complex (miRISC) and promotes DNA damage repair. Their data suggest that this circular RNA contains many sites for binding with a microRNA and promotes the multivalent interactions among miRISCs, which then promotes DNA damage repair by enhancing RAD51 recruitment. Although the underlying mechanisms are unclear, this study provides a novel connection between noncoding RNAs and the proper maintenance of genetic information through phase separation. This research group also wrote a review article [4] on the emerging roles of phase separation in how cells handle DNA double-strand breaks, the most dangerous type of DNA damage. It provides a comprehensive review of the recent reports on how phase separation facilitates the response to DNA double-strand break and the repair process including both homologous recombination and non-homologous end-joining.

The last set of two reviews discuss how phase separation regulates expression of genetic information through transcription. Demmerle et al. [5] focus on transcriptional condensates across spatial, temporal, and genomic scales. They discuss how, at these different scale levels, the transcriptional condensates facilitate transcription factor binding to genomic sites and the activity of RNA polymerase II and regulate responsive 3D chromatin organization. They also discuss the current advances in targeting these condensates for therapeutic purposes and in technical advances that would help further understand these condensates. Mann and Notani [6] also review these transcriptional condensates (called transcription factor condensates in this review), with a unique focus on the dynamic nature of these condensates in serving the rapid transcriptional response to signaling. This review summarizes the protein, RNA, and DNA constituents in the transcription factor condensates and their roles in the condensate function. Using steroid signaling-induced transcription as a model, this paper goes deeply into the formation, maintenance, and dissolution of these condensates in response to signaling, and their functions in regulating signaling-dependent transcription.

This collection is not meant to be complete in covering all nuclear biology. It does not cover phase separation in DNA replication, RNA processing, and many other nuclear events for the proper maintenance and expression of the genetic information. Yet, we hope that the articles in this Special Focus can help readers to better appreciate the essential role of phase separation in accomplishing the central mission of the nucleus.
